# Dual educational rationality and acculturation in Mapuche people in Chile

**DOI:** 10.3389/fpsyg.2023.1112778

**Published:** 2023-03-06

**Authors:** Daniel Quilaqueo, Enrique Riquelme, Darío Paez, María José Mera-Lemp

**Affiliations:** ^1^Facultad de Educación, Universidad Católica de Temuco, Temuco, Chile; ^2^Faculty of Psychology, University of the Basque Country, San Sebastian, Spain; ^3^Facultad de Educación y Ciencias Sociales, Universidad Andres Bello, Santiago, Chile; ^4^Centro Cielo, Facultad de Ciencias Sociales y Comunicaciones, Universidad Santo Tomas, Santiago, Chile

**Keywords:** educational rationality, acculturation, Mapuches, identity, culture

## Abstract

Even though Mapuche people represent the largest indigenous population in Chile, the “logic of double rationality” in their educational knowledge and its link with acculturation dynamics, has been scarcely studied. The aim of this study was to explore the relationships between the attitudes toward school education and the acculturation orientations of 468 Mapuche people, with ages from 17 to 53 years (*M* = 16.19; SD = 7.0). Participants were students of secondary schools and universities from urban and rural areas of different regions of Chile. Results showed that most of the participants presented a bicultural orientation (39.4%), followed by a group of segregated or ethnic identity profile (23.5%), while those who preferred assimilation (17.5%), and marginalization (20.7%) represented a minority. Results indicated that people identified as bicultural scored higher in the components of dual Mapuche/ Chilean Mestizo educational rationality than the other acculturation profiles. Also, the fluency in speaking Mapuche language was positively associated with the perception of cultural differences in knowledge and education between Mapuche and Chilean culture, the evaluation of the teaching of Mapuche culture, the perception of school as a factor of assimilation, the valuation of bicultural practices, and the justification of double rationality. Findings’ contributions to the development of pertinent educational practices in contexts of social and cultural diversity are discussed.

## Introduction

The conceptualization and exploration of acculturation processes have been approached from different perspectives. One of the models, derived from the concept of multiculturalism, is that of bicultural identity where both the identity of the sociocultural mainstream and the sociocultural identity of origin are integrated (Benet-Martínez et al., 2002; Benet-Martínez and Hong, 2014).

However, the adaptive capacity of any given acculturation approach may depend on the prevailing cultural climate in question (Berry, 1997). According to Berry (1980, 1997), Sam and Berry (2010), biculturalism is likely to be adaptive because it allows individuals to interact successfully with both their cultural heritage community and the society in which their cultural group is embedded. Although authors such as Rudmin (2003) argue that biculturalism may be a precarious condition in which the person is rather trapped, pressured both by their home community not to acquire the receiving culture and by the receiving cultural community against retaining the heritage culture (Schwartz et al., 2010).

However, the study of multicultural identities has interesting implications for both psychology and the field of education, as the question of how individuals develop a sense of belonging to a sociocultural group becomes particularly significant in situations of cultural conflict and integration (Baumeister, 1987; Roberts et al., 1999; Karim, 2021). Here, the dynamics of multicultural identity construction provide not only a window to study individual variations in self-concept and social identity development but also an opportunity to explore how the educational rationality of a dominant culture stands as a referent for assessing distance from the knowledge of the sociocultural group of origin. This principle also applies to people who must move between cultures to teach or learn (Sharif, 2019; Stasel, 2021) and need to generate new and richer mechanisms of learning and reflection on their own identity and that of their colleagues/students; that is to say, from the mechanisms of acculturation and biculturality, we can also explore the possibility of constructing a double educational rationality.

### Berry’s model

Berry’s model describes an organizational structure for the search for identity in multicultural contexts; it has conceptualized acculturation by distinguishing processes and outcomes, classifying attitudes and orientations, and categorizing acculturating groups. According to Berry (2017), acculturation strategies are based on two factors that move along a gradient: the desire to maintain one’s own culture and the desire to establish contact with other groups. The dichotomous responses between these two variables will determine the resulting acculturation strategy: integration (maintenance of culture and contact with other groups is important); separation (only contact with other groups is important); assimilation (only maintenance of one’s own culture is important); or marginalization (neither is important). In other words, individuals can be oriented toward the traditional culture, the majority culture, both, or neither. What is not clear, however, is how individuals arrive at such orientations and whether they change over time.

Later, approaches have suggested that the “desire for contact” dimension in Berry’s schema could usefully be replaced by a “desire to adopt the majority culture” (Ward and Kennedy, 1994; Bourhis et al., 1997), and there has been some discussion as to whether these two conceptualizations should be considered synonymous (Snauwaert et al., 2003; Berry and Sabatier, 2010). All in all, the model has remained stable and growing conceptually since its formulation and allows for a dynamic approach to understanding individual and social interaction in socially and culturally diverse settings. Although the model has been frequently applied in interactions between dominant social groups and voluntary minorities (Ogbu, 1991), we consider that it is necessary to explore these dynamics in interactions in which people from indigenous populations represent involuntary social and cultural minorities, and yet maintain their own educational knowledge within the framework of a dominant culture that imposes its own educational logic. Thus, among other elements, the officially recognized language, the educational logic and its contents, and the principles of spirituality are referents of a particular epistemic basis to be considered for potential biculturality and potential double educational rationality. We are not arguing for an essentialist view of culture and the existence of two separate, homogeneous cultures: Mapuche and Chilean. Culture is not a bounded group of people who share practices and beliefs but a box of tools that people use in an adaptive and creative manner (Appadurai, 1988). Culture is a fluid phenomenon. However, shared social beliefs produced by lay people usually use this essentialist view—it is a common point of friction between an anthropologist and indigenous social movements. Urban Mapuche people share an essentialist view of heritage culture, based on homeland and cultural rural features. The claim of an essential culture helps social mobilization in the urban context in which most Mapuche people live (Figueroa, 2015).^1^ Of course, this essentialist view of culture that impregnates social representations of Mapuche collective action plays an ambivalent role. As Brablec posits “While the essentialist connection between identity and the Mapuche homeland, the Wallmapu may appear as a politically required depiction of Mapuche society, it creates a series of obstacles for the majority of the same Mapuche people who currently live in cities. This includes the perpetuation of stereotypes that reduce indigeneity to rural dwelling, anachronistic perceptions of indigeneity as pre-modern in which traditional ways of life are out of place in the fast moving globalized world and the supposed contradiction between indigeneity as local-rural and urbanization as global non-Indigenous” (Brablec, 2021).

Bicultural identity could be conceptualized as dual cultural identity, bicultural integrated identity, and dual cultural adaptation, or bicultural competence. Dual cultural identity refers to the simultaneous and independent development of Mapuche and mainstream cultures. The bicultural integrated approach examines how persons form interaction across the heritage and mainstream culture and include blendedness (overlap vs. dissociation between cultures) and harmony (compatibility vs. opposition). Bicultural persons could share and use two cultures’ rationality in different situations, in a bicultural coexistence or two cultures differentiated and potentially in conflict. In public and work situations, Mapuche probably applies mainstream norms and semantic and procedural knowledge, and in private and primary groups, probably uses Mapuche norms and knowledge—as acculturation studies found in migrants. Another alternative is a mixed or harmonious blending of the two cultures, the creation of a third mestizo culture. Dual cultural adaptation or bicultural competence refers to the ability to jointly access, switch, and use heritage and mainstream culture, this means changes from Mapuche to mainstream frames, norms, and knowledge, and vice versa (Safa and Umaña-Taylor, 2021). The concept of dual rationality, which is developed below, is similar to the last approach (Ward et al., 2018; McAdams et al., 2021).

### Mapuche people and biculturalism

As previously mentioned, the clash between predominant and non-dominant cultures can also be approached from the perspective of native people who, as a result of colonization, are part of a country’s non-dominant social groups and therefore subject to the country’s national culture. The 2017 Census showed (Instituto Nacional de Estadística, 2017) that 12.8% of the population considers itself to belong to some indigenous or native people, of this percentage, 79.8% belong to the Mapuche people, followed by the Aymara people with 7.2%. Due to a sustained emigration from the countryside to the city since the 1930s, there has been a sustained increase in the urban indigenous population over the rural one, which shows 87.8% indigenous members compared to 12.2% living in rural areas. Their migratory processes were taking place strongly after the end, in the early 1930s, of the Chilean occupation of their territories, causing a growing lack of land, which added to the processes of industrialization that the capital was experiencing then, became a pole attraction of thousands of peasant migrants, many of them Mapuche (Campos et al., 2018). Mapuche people live mainly in the Metropolitan Region, mainly in Santiago capital of the nation and a big city of 6 million inhabitants (35.23%), followed by Araucanía (18%) and Los Lagos (12.65%), and to a lesser extent, in the regions of Biobío (9.10%), Los Ríos (5.34%), and Valparaíso (5.31%). The Araucanía, Los Lagos, and Biobío regions are the historical Mapuche rural homeland. Araucanía and Biobío are currently the “heart” of territorial disputes between Mapuche and non-indigenous Chilean (24 Merino et al., 2020). The Mapuche population has higher levels of rurality and poverty. A total of 32% of Indigenous groups live in poverty compared to approximately 20% of Chile’s non-Indigenous population. Poverty is prevalent in the country’s southern rural Indigenous communities (Araucania, Los Lagos, and Biobio) where the Mapuche people live in impoverished enclaves. Indigenous groups have less favorable health indicators than the rest of Chileans. For instance, death from bronchopneumonia in Indigenous children younger than 5 years is proportionally higher than in non-Indigenous children. The rates of tuberculosis, among some Indigenous groups, are double the national average, according to government data, whereas the same applies to child mortality rates. Life expectancy rates are lower in Indigenous communities compared to non-Indigenous groups (Moloney, 2010). In terms of educational quality, Mapuche students achieve lower scores on standardized educational tests and mostly attend schools that serve vulnerable populations, sharing the classroom with low-performing students (Rodríguez-Garcés et al., 2021).

One of the measures taken since the creation of the Chilean State was the establishment of a single monocultural and monolingual education system in Spanish, which denied the use of the Mapuche language and cultural manifestations. Since then, the new generations have learned to live with double educational rationality. Quilaqueo Rapimán et al. (2016) investigate the double educational rationality used by the Mapuche family as a strategy to overcome the monolingual–monocultural Chilean school curriculum. This double rationality is understood as the ability to integrate educational knowledge, which may even be contradictory to different cultural logic. By double rationality, we refer to the coexistence of two “worldviews” or cultural frames, norms, and knowledge, and do not refer to two different types of concepts and cognitive processes. In this sense, double rationality implies a dual cultural competence. The Afro-American sociologist Du Bois (1903) developed a very interesting conceptualization at the beginning of the 20th century; very relevant for people such as the Mapuche (we thank an anonymous reviewer who drew our attention to this author). A person from the discriminated ethnic group feels a duality in his or her self (Dubois twoness): he feels and thinks like an American and an African-American in which two conflicting cultures coexist. In addition, this person experiences discrimination and suffers from the negative evaluation (what Dubois calls the veil) that the dominant culture makes of him (Meer, 2019). In this sense, Dubois shares with another classic Fanon the idea that the dominant culture alienates and humiliates people from subordinate ethnic groups. However, in its development, Dubois suggests that the African–American person can develop a “better” self or self that maintains both cultures and positively integrates them (Meer, 2019). The importance of discrimination is relevant to dual rationality, but we also share that biculturalism can give rise to a person with dual cultural competence and therefore to an integrated cultural identity.

Studies showed that the Mapuche live and coexist with different cultures, and that cultural differences can be categorized from a comparative point of view that varies according to the research question posed. For instance, a study suggests that Mapuche culture stresses emotional self-control. Compared with Chileans, Mapuche people strongly believe that children should overcome fear and be calm and can learn to control emotions by connecting with nature, and by listening and watching elders in the community (Halberstadt et al., 2020).

Thus, to understand the rationality of Mapuche education, it is necessary to bear in mind three central aspects on which it is based. First, an epistemic base of its own culture is sustained by a social memory in relation to how to build knowledge with family and territorial ancestry. Second, the notion of the relationship with knowledge, which aim is to organize the research questions about the Mapuche education. Third, a double educational rationality that has as its axis its own sociocultural knowledge and that which is imposed by the predominant society, which in the case of formal education is the one imposed through the school curriculum of the State of Chile (Quilaqueo, 2012). Although in a simplified way, the Mapuche culture can be synthesized into three topics. First, social relationships are based on respect, which is a central value in social interactions. The values of communication with respect are accentuated when persons are in relation with the elders. Elder condition in the Mapuche culture means a superior status because of the accumulated experience of life and wisdom. Second, Mapuche people have a coexistence relationship with nature based on statements of reciprocity and communication, and nature is conceived as a generous mother who gives the necessary elements for the life of human beings. Third, the relationship with divinity is based on the coexistence and the communication of Mapuche people with each one of the forces that govern their life. Religion is part of daily living and coexists with vital functions such as health and disease. The Machi or indigenous man or woman healer manages the relationship with the divinities and the processes of treatment of diseases.

The education and formation process of the Mapuche children are developed through learning strategies based on oral and attitudinal communication, which involved all the community members. A Mapuche traditional methodology of education is “pentukum” or protocol Mapuche greeting. This method included the development of memory, speech skills, and communication, in general, and then qualities such as prudence, empathy, solidarity, and respect among others. Since childhood Mapuche are sent as “Werken” or messengers to visit close relatives, children had to transmit information, as their mother or father, or any other relatives requested it. Another method is Nütram or Conversations. For Mapuche people, the conversation is an important part of oral communication among people and children, they had to understand the importance of conversation, talking, dialoguing, not only listening, and they had to speak to parents or other relatives or members of the community in an atmosphere of trust-developed learning. Another educational method is Gülam or advice by elders. The epistemic basis of Mapuche education is also supported by the concept of kimeltuwün, as the methodological framework of education based on the social construction of knowledge imparted by parents (Quilaqueo, 2015). This educational logic guides the construction of knowledge with family and territorial ancestry, supported by the social memory of the family and its community with the historical Mapuche (Quilaqueo and Quintriqueo, 2017), and includes teaching the attributes that the Mapuche person should have, such as previously mentioned respect, solidarity, and esteem for other members of the community.

The notion of relationship with knowledge has to do with the construction of discourse-based knowledge from the social memory and orally communicated in the family context (Montesperelli, 2004; Quilaqueo, 2019). A different relationship with knowledge is created depending on whether it is about training as a person or specific content for the training of a job or work role (Alonqueo, 1985; Quilaqueo, 2019).

Following Lahire’s equation (Lahire, 2012) in which the incorporated past + present action context = gives rise to observable practices, the double educational rationality from Mapuche knowledge is the result of past social practices in educational action based on the social memory of family educational discourses and integration to the Western educational scenario in the present action context. Thus, there is evidence of observable social practices of double educational immersion that consider the Mapuche family and a monocultural education from the non-Mapuche society—Chilean mestizo culture. In this double rationality, it is fundamental to take into account that the incorporated past is linked to a “family inheritance” of a “knowledge of belonging,” the küpan where it is incorporated, for example, the perception of “loss of meaning” and of belonging to the Mapuche historical collectivity (Curivil, 2007).

Social mobilizations during the recent decade raised and succeeded in imposing Intercultural Bilingual Education (IBE). An intercultural bilingual education program was created late in 1994. The law establishes that if there is a minimum of 20% Mapuche students, a bilingual education must be carried out. It is important to remark that the Mapuche parents have unilaterally developed this educational double immersion because they are the ones who have rationalized the social and cultural clash at school as social actors.

Qualitative studies describe how this dual rationality logic could be displayed—at least partially. An ethnographic study illustrates how Mapuche indigenous knowledge (Kimün) and language (Mapudungun or Mapunzugun) were incorporated into an Intercultural Bilingual Education (IBE) program of a school within a Mapuche context in Chile. Based on a 6-month school ethnography, this study shows the role of an ancestral educator (Kimche) who, as a teacher in the IBE program, becomes an agent of Indigenous cultural and linguistic transmission as he brings Indigenous knowledge into the classroom as his main curricular objectives (Ortiz, 2009). Another article, based on two school ethnographies, analyzes Mapuche language and culture teaching practices of a rural school in the Chilean Araucanía region run by Mapuche teachers, aimed at reinforcing the language, culture, and identity of children. The study describes the ways children learn to be Mapuche, by participating in social and communitarian practices reproduced in school. While the teaching of mainstream culture and school contents are conducted in standard ways, Mapuche language and culture are transmitted and learned through the recreation of modes of living, producing, and reproducing indigenous traditional knowledge that normally occurs in family and community (Luna et al., 2018).

In summary, Mapuche education has its own characteristics that differ from Western education in terms of the fact that it is based on different logic for the formation of people. In a scenario of domination, Mapuche education has had to integrate the logic of Western (Chilean) education, but it has kept its own logic on the margins of school education.

A meta-analysis found a significant association between proficiency in heritage or ethnic language and the sense of ethnic identity (Mu, 2015). Our previous qualitative studies also suggest the relevance of language skills and use as a factor that contributes to the maintenance of the family memory and enhances belonging to the Mapuche history and collective and allows it to fix its own knowledge from where differences between Mapuche and Chilean school educational knowledge are acknowledged.

Suggesting that biculturalism is the dominant identity, a survey conducted in the Araucanía area found that 45% of persons considered themselves both Mapuche and Chilean, 28% considered themselves Mapuche or mostly Mapuche, 17% considered themselves only Chilean or mostly Chilean, and 1% did not answer (Le Foulon, 2022).

Previous studies show that Mapuche ethnic collective identity was related to reported higher discrimination, to support the Mapuche social movement, and to higher wellbeing, as well as the moderate negative impact of discrimination on wellbeing (García et al., 2021). Mapuche ethnic identity has also been related to low levels of antisocial behaviors (Jiménez Bustos et al., 2017).

### The present investigation

The results obtained by the study of Quilaqueo Rapimán et al. (2016) lead to the question of whether Mapuche’s educational double rationality can be evaluated under the prism of acculturation as proposed in Berry’s model (Berry and Sabatier, 2010). This research aimed to explore the association between the dual rationality strategy in Mapuche education and the four categories of acculturation strategies in Berry’s model. It is expected that bicultural and ethnic identity should be related to H1—fluency in speaking Mapunzugun or Mapuche language; H2—a higher perception of cultural differences in knowledge and education between Mapuche and Chilean culture, a more positive evaluation of the teaching of Mapuche culture, the perception of school as a factor of assimilation, the valuation of bicultural practices, and the justification of double rationality. It is expected to find the opposite in the case of assimilation and marginalization H3—fluency in the heritage language or Mapuche will show a specific influence on the perception of differences between Mapuche and Chilean culture.

## Methodology

### Participants

A total of 468 people participated, ranging in age from 17 to 53 years (*M* = 16.19; SD = 7.0): men (43%) and women (57%); elementary (43.4%), middle school (30.6%), and high school (21.1%) students; from urban (57.5%) and rural (42.5%) sectors; from the Metropolitan (19.2%), La Araucanía (54.7%), and Los Lagos (26.1%) regions. The participants were chosen through a non-probabilistic sampling, by accessibility, from schools, high schools, and universities in the aforementioned regions. All agreed to participate voluntarily and in the case of minors with parental consent. Probably because we rely on voluntary responses, our sample will be biased toward people sharing a more reflective and sensitive attitude toward the Mapuche issue. Our sample has an overrepresentation of the historic rural areas of the Mapuche homeland such as Araucanía—although most people live in urban areas.

### Procedure

This study was part of a larger project, where university academics and school teachers (both Mapuche and non-Mapuche) from the different regions mentioned were part of the research team, they organized themselves to generate a previous link with the schools and families of those who answered the survey, being able to not only explain the purposes of the study, roles, receive queries, comments, and suggestions but also to feedback the results; this process was also approved by the research ethics committee of the Catholic University of Temuco (2018–2020).

The survey was applied during 2018–2019. An area coordinator (for example, for an Araucanía town hall or community) contacted the high school and university teachers who applied the questionnaires to the students in mass classes. The students collected questionnaires from their parents, after having answered them and clarified any doubts they had. The instrument was applied on paper and pencil. There is no payment for answering the questionnaires. The response rate was very high in the classes, and in the case of the parents, it was 40%.

### Instruments

#### Cultural orientation

To evaluate the acculturation strategies of Mapuche participants, two items based on Berry’s (2017) Acculturation Model were used: one on attitude toward Mapuche culture (“Mapuche people should try to live according to their customs”) and another toward the dominant culture (“Mapuche people should try to participate fully in the life of non-Mapuche society in Chile”). Participants were divided based on the theoretical mean: on a scale of 1–5, scores of 4 and 5 were categorized as high and scores of 3 or less were categorized as low. Those with high scores on both questions are conceived as “bicultural,” and those with high scores in Mapuche culture maintenance and low in Chilean culture as “segregated, separated,” or ethnic identity, those with low responses in Mapuche culture maintenance and high orientation toward Chilean culture as “assimilated,” and those with low scores on both dimensions as “marginal.”

#### The scale of attitudes toward school education

This scale was developed from the model proposed by Quilaqueo et al. (Figueroa, 2015), to explore implicit models of the oral social memory of historical Mapuche collectivities that in contexts of present action imply particular practices at the educational level, both from the traditional and the predominant Western model. The scale is composed of 30 Likert-type items with five response options (1 = Strongly disagree and 5 = Strongly agree), which inquire into five dimensions conceptualized from the theoretical framework of double rationality as follows:

- *Perception of cultural differences in knowledge and education* between the Mapuche and Chilean cultures (“In general, the Mapuche culture has different knowledge and education from the Chilean culture,” “in general, Mapuche and non-Mapuche people differ in the way they think,” “in general, Mapuche educational logic teaches more respect for nature than school logic”; *α* = 0.70; *Ω* = 0.70);
- *Importance of* teaching *Mapuche culture for integration of knowledge and identity* (e.g., “I think that Mapuche knowledge should be taught at school,” “it would be necessary to teach the Mapuche language at school to strengthen Mapuche identity”; *α* = 0.71; *Ω* = 0.70);
- *Perception of the school as an assimilating factor* (e.g., “In general, the Mapuche educational logic teaches to be more independent than the school’s rationality,” “the school’s education transmits to the Mapuche the idea of being Chilean and not Mapuche”; *α* = 0.61, *Ω* = 0.62);
- *Justification of the double rationality*, which inquiries into the perception about the effects of schooling and coexistence on the need for assimilation of the Mapuche, as well as the resilient effect of maintaining the Mapuche language and cultural thinking (e.g., “What I learned at school prevents me from developing my identity because I was not taught the Mapuche language,” “when I speak in Mapunzugun I think better as Mapuche”; *α* = 0.66, *Ω* = 0.62).
- *Positive valuation of bicultural practices* (e.g., “I would like children to be trained in both knowledge: Winka and Mapuche,” “people who have Mapuche and non-Mapuche knowledge are wiser: they have two wisdoms”; *α* = 0.59, *Ω* = 0.60).

The results of the confirmatory factor analysis of five factors were satisfactory: *χ*^2^ (340) = 730.389, *p* < 0.001; CFI = 0.827; TLI = 0.878; SRMR = 0.057; RMSEA = 0.050 (90% CI [0.045, 0.054]).

### Statistical analysis

Analysis using crosstabs and Chi-square (*χ*^2^) was carried out regarding the relationship of acculturation typologies with the sociodemographic characteristics of the participants, such as rural vs. urban context, educational level, age, and sex. To explore the association between urban vs. rural context, age and sex with dimensions of attitudes toward school education, Pearson and point-biserial correlation analyses were used to study the associations between the aforementioned sociodemographic variables, and the dimensions of attitudes toward school education.

To test H1 and H2, omnibus analysis of variance or ANOVA was carried out using Berry’s categorical model (acculturative typologies) as fixed variables and dimensions of the dual educational rationality model as a dependent variable. To examine the H3 correlation between dual rationality scale dimensions and variables age, urban, schooling, and linguistic fluency in Mapunzugun were carried out. To examine the specific influence of fluency in speaking Mapunzugun as a predictor of dual rationality dimensions, multiple regression analysis was carried out — controlling or using as a simultaneous predictor age, urban, schooling, and sex.

For a probability of a Type I error of *α* = 0.05 and a probability of a Type II error (1–*β*) of 0.80, and for an effect size of *r* = 0.20 or *d* = 0.40, the sample size is *N* = 194—for a one-sided test (Rosenthal and Rosnow, 2008). The sample size is satisfactory for statistical power.

## Results

### Typologies of identity and culture

**Figure 1:**
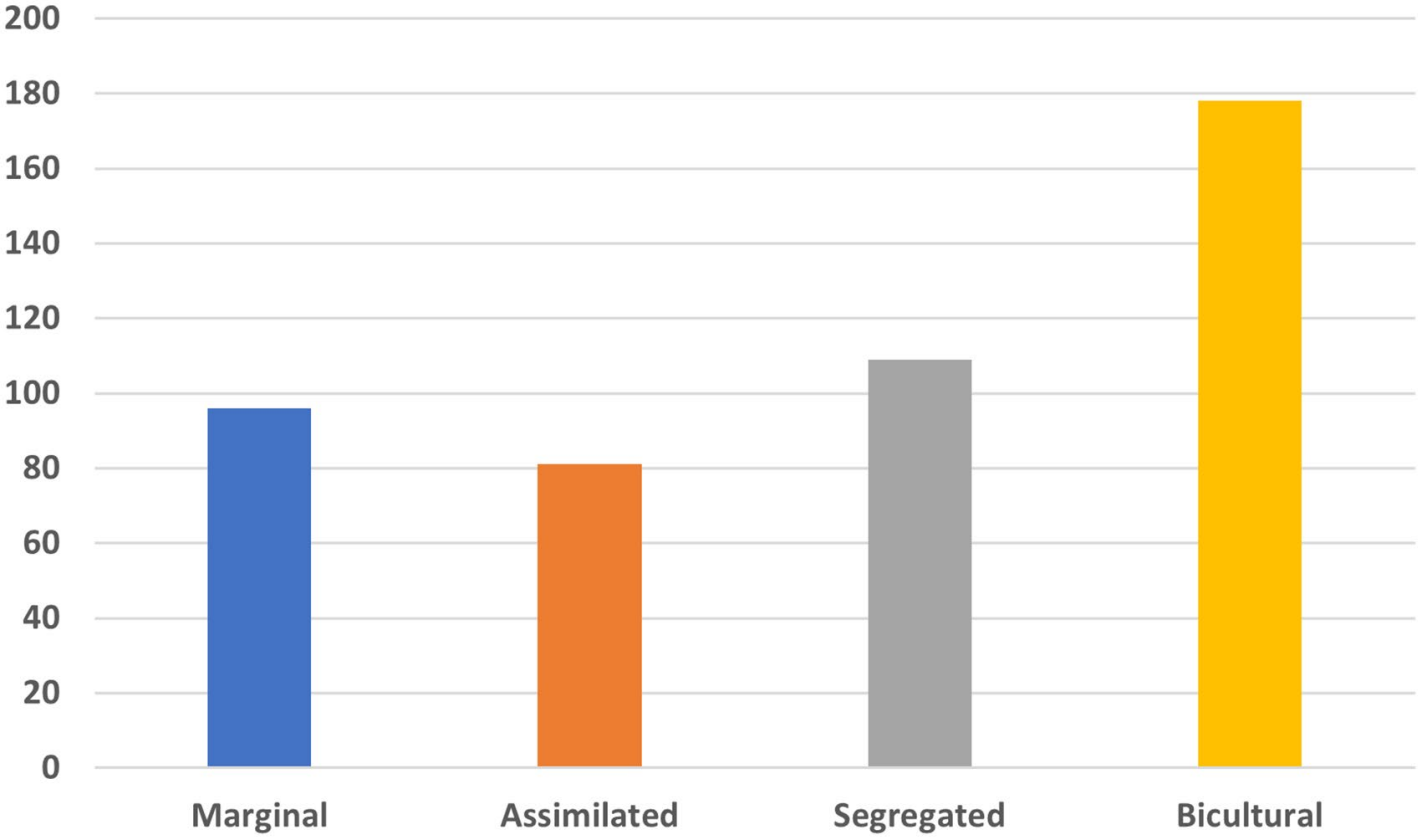
Acculturation orientation: marginal 21%, assimilated 17.5%, segregated 23.5%, bicultural 39.4%.

Regarding the acculturation orientations of the participants, it was found that a minority corresponded to the “marginal” profile and the “assimilated” group. The “ethnic identity” or “segregated” group was a larger minority. Finally, bicultural people were the largest group (see Figure 1).

Participants’ answers to the two items about their attitudes toward Mapuche culture and the dominant culture, showed that the orientation to maintain the Mapuche culture predominated (61.9% agreement), while the agreement with the integration into the Chilean society was lower (55.8%).

**Figure 2:**
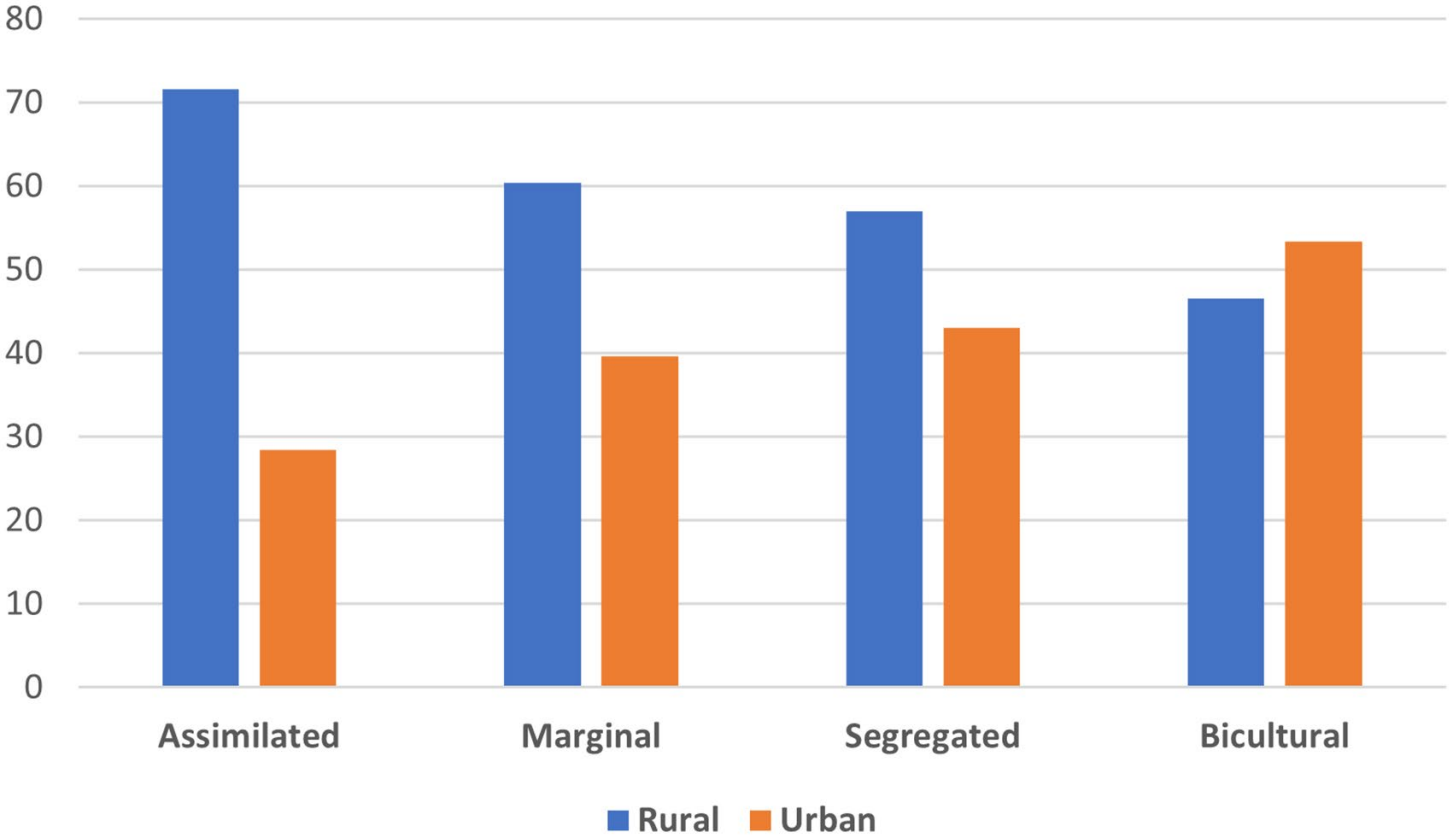
Percentage of acculturation orientation by urban and rural context.

The analysis was carried out regarding the relationship of these typologies with the sociodemographic characteristics of the participants, such as rural vs. urban context, educational level, age, and sex. It was found that there is a significant association between rural/urban context and acculturation profile (*χ*^2^ (3,464) = 15.150; *p* < 0.01). Most of the participants of the assimilated, marginal, and ethnic identity profiles belong to rural settings (71.6, 60.4, and 57%, respectively). In relation to the bicultural typology, there is a slightly higher proportion of urban participants (53.4%; see Figure 2).

Significant associations were also found between the acculturation profiles and the educational level of the participants (*χ*^2^ (6,464) = 18.439; *p* < 0.01). It stands out that the presence of bicultural participants is higher as educational level increases and that ethnic identity and non-identified remain as two relatively stable minorities, while assimilated decreases as educational level increases. On the other hand, a positive association was found between age and biculturality (*r* = 0.20; *p* < 0.01). This suggests that as age increases, there is a higher articulation of identification with Mapuche culture and the Chilean majority culture.

There are no significant associations between the sex of the participants and the acculturative typology to which they belong.

### Typology differences in language fluency

To test H1 and H2, ANOVA was carried out using Berry’s categorical model (acculturative typologies) as fixed variables and dimensions of the dual educational rationality model as dependent variables (Table 1).

**Table 1:** Means, standard deviations, and One-Way analyses of Variance in the dimensions of attitudes toward school education.

Measure	Acculturative typology	*N*	*M*	SD	*F* (3,463)	*p*
Cultural differences in knowledge and education	Marginalization	96	3.41	0.783	19,458 ^acdf^	0.000
	Ethnic identity	109	3.94	0.716		
	Assimilation	81	3.66	0.804		
	Biculturalism	178	4.07	0.668		
	Total	464	3.83	0.771		
Importance of teaching Mapuche culture for integration of knowledge and identity	Marginalization	96	3.64	1.085	12,547 ^cef^	0.000
	Ethnic identity	109	3.90	0.976		
	Assimilation	81	3.77	0.908		
	Biculturalism	178	4.29	0.794		
	Total	464	3.97	0.957		
Perception of the school as an assimilating factor	Marginalization	96	2.87	0.850	10,910 ^acf^	0.000
	Ethnic identity	109	3.26	0.894		
	Assimilation	81	2.99	0.859		
	Biculturalism	178	3.44	0.894		
	Total	464	3.20	0.907		
Justification of the double rationality	Marginalization	96	3.02	0.756	17,127 ^acf^	0.000
	Ethnic identity	109	3.50	0.721		
	Assimilation	81	3.30	0.800		
	Biculturalism	178	3.70	0.765		
	Total	464	3.44	0.798		
Positive valuation of bicultural practices	Marginalization	96	3.26	0.707	15,895 ^cef^	0.000
	Ethnic identity	109	3.41	0.845		
	Assimilation	81	3.51	0.739		
	Biculturalism	178	3.89	0.727		
	Total	464	3.54	0.787		

Duncans’ *post-hoc* test: ^a^significative difference between Marginalization and Ethnic Identity, ^b^significative difference between Marginalization and Assimilation, ^c^significative difference between Marginalization and Biculturalism, ^d^significative difference between Ethnic Identity and Assimilation, ^e^significative difference between Ethnic Identity and Biculturalism, ^f^significative difference between Assimilation and Biculturalism.

In the fluency in speaking Mapunzugun, an omnibus ANOVA found significative differences between identity types (*F* (3,463) = 19.458; *p* < 0.001). Bicultural and ethnic identity groups score higher in fluency in speaking Mapunzugun than assimilated and marginalized groups.

### Typology differences in the perception of cultural differences

In the dimension of perception of cultural differences in knowledge and education between Mapuche and Chilean culture, results showed significative differences (*F* (3,463) = 19.458; *p* < 0.001). According to *post hoc* Duncan test, the bicultural group scored higher (*M* = 4.07) than the assimilated (*M* = 3.66) and marginalized (*M* = 3.41) groups. People of ethnic identity typology also rated higher (*M* = 3.94) than the assimilated and marginalized groups in this dimension. There was no difference between bicultural and ethnic identity group participants. All groups scored above the theoretical mean, and the difference was graded rather than extreme. Taking the extreme scores (4 and 5) as indicators of strong agreement with this scale, it was found that 68.5% of the bicultural group and 51.4% of people with high ethnic identity shared these ratings, compared to only 39.5% of assimilated and 27.1% of marginalized people.

### Typology differences in the importance of teaching Mapuche culture

In the dimension of the importance of Mapuche cultural teaching for the integration of knowledge and identity, significant differences were found (*F* (3,463) = 12.547; *p* < 0.001). Bicultural people also score higher (*M* = 4.29) than all the other groups. There were no differences between people of high ethnic identity (*M* = 3.90), assimilated (*M* = 3.77), and marginalized (*M* = 3.64) groups. Taking the extreme scores (4 and 5) as indicators of strong agreement with this scale, these were found in 77% of the bicultural and 58.7% of the high ethnic identity people vs. 53.1% of the assimilated and 49% of the marginalized people.

### Typology differences in the perception of school as a factor of assimilation

In the questions on school as a factor of assimilation, significant differences were also found (*F* (3,463) = 10.91; *p* < 0.001). Bicultural people also score higher (*M* = 3.44) than the marginalized (*M* = 2.86) and assimilated (*M* = 2.99) groups while those of ethnic identity (*M* = 3.26) score higher than the marginalized group. Taking the extreme scores (4 and 5) as indicators of strong agreement with this scale, these were found in 42.7% of bicultural and 35% of persons of high ethnic identity vs. only 22.1% of assimilated and 9.8% of marginalized people.

### Typology differences in the justification of dual rationality

Significant differences were found between groups in the dimension of justification of dual rationality (F*F* (3,463) = 17.127; *p* < 0.001). Bicultural people scored higher (*M* = 3.70) than marginalized (*M* = 3.02) and assimilated (*M* = 3.30) groups, and ethnic identity (*M* = 3.50) scored higher than marginalized groups. Taking the extreme scores (4 and 5) as indicators of strong agreement with this scale, it was found that 55.7% of bicultural people and 26% of participants with a high ethnic identity strongly justify assimilation as an effect of contact with the dominant majority and defend the maintenance of Mapunzugun, compared to 11.5% of assimilated and 6.9% of marginalized people.

### Typology differences in the positive evaluation of bicultural practices

In the positive evaluation of the bicultural practices dimension, omnibus ANOVA found significative differences (*F* (3,463) = 15.895; *p* < 0.001). Bicultural group scores were higher (*M* = 3.89) than the Mapuche ethnic identity groups (*M* = 3.41), the marginalized (3.26), and the assimilated (*M* = 3.51) groups. However, all groups scored above the theoretical mean, and the difference is graded and not extreme. Taking the extreme scores (4 and 5) as indicators of strong agreement with this scale, it was found that 52.6% of bicultural and 30.4% of people with high ethnic identity share these ideas, compared to 36.7% of assimilated and 16.5% of marginalized people.

In addition, identification with Mapuche but also with Chileans correlates positively with all dimensions, suggesting that identification with ethnic group reinforces agreement with perceived cultural differences but that national identity is not an inhibitory factor to recognize it.^2^

### Correlation analysis between fluency in speaking Mapunzugun or Mapudungun, urban vs. rural context, and age with attitude *toward* education

To examine H3, correlation analysis was carried out. Results showed that the higher fluency in speaking Mapunzugun was positively associated with all dimensions of attitudes toward school education: perception of cultural differences in knowledge and education between Mapuche and Chilean culture (*r* = 0.30; *p* < 0.001), valuation of the teaching of Mapuche culture (*r* = 0. 30; *p* < 0.001), perception of school as a factor of assimilation (*r* = 0.30; *p* < 0.001), valuation of bicultural practices (*r* = 0.29; *p* < 0.001), and the justification of double rationality (*r* = 0.24; *p* < 0.001).

To explore the association between urban vs. rural contexts, point-biserial correlations were carried out. The results also revealed that belonging to an urban environment (urban = 1; rural = 2) was associated with a higher perception of cultural differences in knowledge and education between Mapuche and Chilean culture (*r* = −0.29; *p* < 0.001), a higher valuation of the teaching of Mapuche culture for the integration of knowledge and identity (*r* = −0.24; *p* < 0.001), higher perception of school as a factor of assimilation (*r* = −0.40: *p* < 0.001), higher justification of dual rationality (*r* = −0.21; *p* < 0.001), although not with a higher valuation of the positive effects of bicultural practices. In addition, as the age of the participants increased, the difference in the perception of the school as an assimilating agent (*r* = 0.37; *p* < 0.001), the perceived cultural difference (*r* = 0.27; *p* < 0.001), the valuation of the teaching of Mapuche culture (*r* = 0.18; *p* < 0.001), the justification of the double rationality (*r* = 0.18; *p* < 0.001), and the practices that integrate both cultures (*r* = 0.17; *p* < 0.001) were also increased. No associations were found between the sex of the participants and the dimensions studied.

### Multiple regression analysis for the specific influence of fluency in speaking Mapunzugun or Mapudungun

To examine H3 on the specific influence of each variable, regression analyses were run on the scale dimensions for the predictor’s age, urban, schooling, and linguistic fluency in Mapunzugun. Controlling for the influence of educational level, in order of importance, participants fluent in Mapunzugun (*β* = 0.16; *p* < 0.001) and of higher age (*β* = 0.03; *p* < 0.05), and to a lesser extent, from urban contexts (*β* = −0.19; *p* < 0.05) perceive more differences between Mapuche and Chilean culture (*F* (4,467) = 17.698; *p* < 0.001).

To examine the effects of linguistic fluency in Mapunzugun on each dimension of the scale, regression analyses were run. (*β* = 0.16; *p* < 0.001) and of higher age (*β* = 0.03; *p* < 0.05), and to a lesser extent, from urban contexts (*β* = −0.19; *p* < 0.05) perceive more differences between Mapuche and Chilean culture (*F* (4,467) = 17.698; *p* < 0.001).

Similarly, controlling for the influence of educational level and age, people who speak fluently Mapunzugun (*β* = 0.22; *p* < 0.001) and to a lesser extent from an urban environment (*β* = −0.23; *p* = 0.058), value Mapuche education more highly (*F* (4,467) = 13.862; *p* < 0.001).

It is also observed that controlling for the influence of age, environment, and educational level, those who are more fluent in Mapunzugun (*β* = 0.14; *p* < 0.001) perceive largely that the environment forces them to assimilate (*F* (4,467) = 9.176; *p* < 0.001). Finally, controlling for the influence of educational level, age, and environment, people who speak the Mapuche language more fluently (*β* = 0.19; *p* < 0.001) value bicultural practices more highly (*F* (4,467) = 10.933; *p* < 0.001). Results support that fluency in heritage language reinforces all dimensions of attitudes toward school.

## Discussion

Descriptive results revealed that most of the participants presented a bicultural profile, representing 39.4% of the total sample. This distribution has been previously reported in Chile (see, Le Foulon, 2022) for surveys in 2006 and 2016 showing the importance of bicultural identity and in the context of Sweden and Norway with Sami minorities, living in these countries, 32% ethnic identity, 32% bicultural, and 36% assimilated were found (Kvernmo and Heyerdahl, 1996). In a zone of cultural contact, a “blended” culture usually emerges. Our data suggest that the last one is the more prevalent because double identification was the more common answer—not only in our sample but also in a general survey. For instance, members of urban Mapuche associations tended to self-identify as Mapuche champurria (the mixture of different things without a unique form in Mapunzugun) or sharing an identity based on cultural hybridization, evidencing their cultural “mestizaje” (Brablec, 2021). “Champurriado(a”) is a person who is part Mapuche and part Chilean. This mixed identity is evidenced many times in the surnames (Mapuche and Castillian simultaneously). The champurria is a person who has two worlds. It is someone in whom two cultures inhabit (Moraga, 2021). An important minority reports a Mapuche identity or segregated or purely ethnic identity. As a strategy of resistance to assimilation, not only rural but also urban Mapuche have tended to foster conceptions of ethnocultural purity to defend their threatened cultures (Brablec, 2021). Our results are congruent with findings that indigenous migrants in cities are strongly oriented toward not only maintaining their culture, continuing their indigenous collective identity, mainly by biculturalism but also by maintaining an indigenous ethnic identity (Campos et al., 2018).

Among our sample, a minority think of themselves as sharing a Chilean identity. In that sense, they are “awinkado,” a person who challenges the Mapuche culture and begins to behave according to what the Mapuche conceive as Winka (white mestizo Chilean) cultural codes (Brablec, 2021). Finally, marginal orientation was relatively important. In fact, studies suggest that a proportion of urban Mapuche have had to learn to live as outsiders in both rural and urban worlds. They face pressure from the non-Indigenous urban society to hide their indigeneity and from the rural Mapuche society of origin who accuse them of a lack of authenticity (Brablec, 2021). In this sample, a great typological variability could be observed within a framework of high identity with Mapuche culture and, similarly, although with less strength, identification with Chilean culture.

Results supported H1, indicating that participants with high Mapuche ethnic identity and bicultural identity were more fluent in Mapuche language. However, it is important to be aware that fluency in Mapudungun is limited since a survey found that among self-identified Mapuche respondents, 76% of urban Mapuche people and 57% of rural Mapuche people did not speak Mapuche (Le Foulon, 2022). In fact, approximately 10% of the Chilean population belongs to an indigenous group, but only 1–2% speak Mapunzugun and other indigenous languages.

They also supported H2 with nuance. Bicultural and those who share a Mapuche ethnic identity agree more than the assimilated and marginalized people in the perception of cultural differences in knowledge and education between Mapuche and Chilean culture. Bicultural and ethnic identity participants also agree more on school as a factor of assimilation, as well as agree more with the justification of dual rationality than marginal identity—and bicultural identity also score higher than assimilated in these two dimensions. However, on the importance of Mapuche cultural teaching for the integration of knowledge and identity and positive evaluation of bicultural practices, bicultural participants score higher than the other three groups. Bicultural participants show the greatest number of differences, in addition to being those who most value bicultural practices, which is to be expected and validates this typology. Similarly, they are the ones who most insist on teaching Mapuche culture at school, showing a more proactive attitude than those with an ethnic identity. We have been able to observe that bicultural people with double educational rationality are those who most value the knowledge of Mapuche culture and those practices that seek the integration of both cultures. Bicultural people, from a perspective of double educational rationality, assume and value their own Mapuche sociocultural knowledge, which is contrasted with Chilean culture. They do not reject the knowledge imposed by the dominant culture.

Finally, both identification with Mapuche and Chilean people were positively associated with the perception of differences in knowledge and education between Mapuche and Chilean culture, the valuation of the teaching of Mapuche culture, the perception of the school as an assimilating agent, the justification of the double rationality, and the practices that integrate both cultures. The association was strong with the identification with Mapuche people than with Chilean people as could be expected, but suggest that identification with a national group is not an obstacle and even reinforces the perception of cultural differences and the importance of Mapuche culture.

One of the key elements in the development of bicultural people who have developed a double educational rationality has been the mastery of the Mapuche language: Mapunzugun. In fact, the higher the fluency in speaking Mapunzugun, the greater the perception of cultural differences in educational knowledge between Mapuche and Chilean culture, the more the teaching of Mapuche cultural elements is valued, the more the school is perceived as a factor of assimilation, the more bicultural practices are valued, and the more the context is perceived as forcing adaptation. Indeed, even controlling for the influence of age and level of schooling, people with higher fluency in the use of Mapunzugun perceive more differences between Mapuche and Chilean culture and the school as an assimilating agent, which the social context forces them to adapt to, and they value to a greater extent both education based on Mapuche culture and practices that integrate both cultures. Supporting H3, the linguistic factor, thus, reinforces both beliefs and behaviors of self-recognition and defense of historical collective knowledge, as well as behaviors that proactively seek identity without harming an orientation toward bicultural practices where Western knowledge is predominant.

Exploratory analysis of the relationships between the sociodemographic characteristics of the participants and the dimensions studied found that belonging to an urban environment is associated with a higher perception of differences between Mapuche and Chilean culture, a higher valuation of Mapuche education and culture, a higher perception of school as a factor of assimilation and coherently with a higher view that the environment forces assimilation and adaptation. This suggests that daily contact with Chilean culture reinforces the perception of differences and the pressure toward acculturation. Overall, a more critical view characterizes people living in urban environments. We emphasize that these people are older or are receiving a university education. However, the differences described are maintained when comparing only people with basic and middle school education. A survey of Mapuche secondary school students, residing in the urban area of Santiago (Region Metropolitana) and Temuco (Araucania), confirms that the main tendency among them is not only to maintain the original culture [average agreement of 5.2 on a scale of 1 (not at all) to 7 (a lot)] but also to adopt the Chilean mainstream culture (4.8 out of 7), as well as identification as medium–high Mapuche (4.8 out of 7). The agreement to maintain the Mapuche culture strongly correlated with the acceptance of the Chilean culture, showing that they are not two opposite options and that Mapuche students opt for biculturalism. In addition, these scores correlated when measured at an interval of 6 months, showing that they were stable (González et al., 2017). Globally, these results support the prevalence of biculturalism and double rationality among urban Mapuche people.

From a double educational rationality point of view, the results allow us to observe that people who have scored higher in the ability to recognize and integrate educational knowledge from different cultural rationalities and biculturality are also more able to identify differences between western educational knowledge and their own, as well as to evaluate the school and school knowledge as an assimilating agent, the above is also associated with the perception that the context forces to adapt and acculturate (Quilaqueo, 2012). The double rationality would then imply the development of a double educational immersion (Quilaqueo Rapimán et al., 2016), which could force the recognition of social and cultural shock at school.

Thus, from Lahire’s (2012, 2017) model, the present context of action is then graphed by the recognition of a specific position from where it is possible to appreciate the disdain for Mapuche educational knowledge by the dominant culture, as well as the pressure to exclude their own knowledge from the equation in the double rationality/biculturality.

On the other hand, the specific influence of the urban context only emerges clearly in the dimension of the school as an agent of assimilation, and marginally in relation to the perception of cultural differences and the valuation of knowledge of Mapuche culture. Qualitative studies suggest that ethnic identity appears to be strongly linked to an individual’s schooling process. Moments of cultural awakening are observed, first, when schooling begins in second infancy; second, entering adolescence and starting secondary school; and finally, at the start of higher education. In the case of Mapuche adolescents, some studies have reported that the perception of threats to their ethnic identity is linked to high levels of stress and negative psychological outcomes. However, the development of ethnic identity is also linked to feelings of wellbeing, suggesting that the school experience has an ambivalent effect (Zañartu et al., 2021).

To conclude from Lahire’s model (Lahire, 2012, 2017), we can then contribute to the biculturality model by making some precisions from the double rationality as an explanatory mechanism: incorporating past (Mapuche knowledge through the mastery of Mapunzugun) + present context of action (recognition of the relative position of own knowledge in relation to Western knowledge) = observable practices of recognition and articulation between own and Western knowledge at school.

It is important to be aware that even if intercultural and bilingual programming exists, some scholars have a critical view of them. Intercultural Bilingual Education Program in Chile aims to improve the quality and relevance of learning from curricular contextualization, to teach indigenous culture, traditions, and languages to children and youth Mapuche. Following Karim, “A culture learning approach to the study of acculturation can help address the existing gaps, extend the theory, draw contextualized conclusions, and take appropriate steps in education to prepare younger generations for an interconnected and interdependent world” (Karim, 2021).

However, the lack of support for traditional educators and the decontextualized curriculum have limited their positive impact. These bilingual programs do not decrease differences and conflict between native peoples’ languages and Chilean mainstream culture (Santiagos and Quiroga-Curín, 2022). Another criticism is that a new “color blind” and “color dumb” racism is embedded in educational systems—and reproduced by teachers and peers in schools. New racism is characterized by teachers’ ‘racelessness’ approach that denies ongoing racism and underpins mainstream cultural norms and microsocial practices of structural racism. The experiences of Chile’s Mapuche youth reflect the damaging outcomes of not talking about race and racism and the limits of intercultural bilingual education. This racism thrives in new ways in Chilean schools, with negative effects on some indigenous youth’s personal and collective identities.

Lahire’s (2012, 2017) proposal for double rationality would imply a possibility to partially explain biculturality with the context of present social action and reference to the past through language with a framework of perceived loss of meaning in Mapuche educational double rationality.

## Data availability statement

The raw data supporting the conclusions of this article will be made available by the authors, without undue reservation.

## Ethics statement

The studies involving human participants were reviewed and approved by Comité de ética de la Investigación de la Universidad Católica de Temuco, Chile. Written informed consent to participate in this study was provided by the participants’ legal guardian/next of kin.

## Author contributions

ER and DQ designed the study and collected the data. DP and MM-L carried out the data analysis. ER, DQ, DP and MM-L collaborated in interpretation of the data, drafted the manuscript and discussed the results. All the authors contributed to the article and approved the submitted version. 

## Funding

Fondecyt Regular No. 1181314 “Diálogo de saberes educativos mapuche y escolar: construcción de una base epistémica intercultural de conocimientos”; Fondecyt Regular No. 1191956 “Educación escolar: socialización emocional en contextos de diversidad social y cultural” and Fondecyt No. 1231178 “Ambivalencia sociocultural y educativa en contexto mapuche: tensión epistémica de docentes con estudiantes y padres de familia”.

## Conflict of interest

The authors declare that the research was conducted in the absence of any commercial or financial relationships that could be construed as a potential conflict of interest.

## Publisher’s note

All claims expressed in this article are solely those of the authors and do not necessarily represent those of their affiliated organizations, or those of the publisher, the editors and the reviewers. Any product that may be evaluated in this article, or claim that may be made by its manufacturer, is not guaranteed or endorsed by the publisher.

## Footnotes

^1^An etnographical study in a rural school concludes: "The rural school studied is a charter school located in the Araucania Region, and is defined by its teachers as "non-intercultural." This educational center has appropriated the school space in order to develop a project of "Mapuche education" linked to a political agenda of selfdetermination…despite the anti-intercultural and essentialist discourses, the school is a community of practice where different elements and meanings coexist in a bicultural logic. This bicultural coexistence and the predominance of the traditional school model represent for children a significant knowledge background that counterbalances teachers’ work on raising consciousness about being Mapuche. The functionality of an essentialist vision of Mapuche culture aimed at social and political empowerment is discussed" (p. 1).

^2^Mapuche identification correlates with the perceived cultural difference (*r* = 0.32; *p* < 0.001), the valuation of the teaching of Mapuche culture (*r* = 0.23; *p* < 0.001), with the perception of the school as an assimilating agent (*r* = 0.24; *p* < 0.001), the justification of the double rationality (*r* = 0.28; *p* < 0.001), and the practices that integrate both cultures(*r* = 0.25; *p* < 0.001), Chilean identification also correlates with the perceived cultural difference (*r* = 0.16; *p* < 0.001), the valuation of the teaching of Mapuche culture (*r* = 0.18; *p* < 0.001), with the perception of the school as an assimilating agent (*r* = 0.13; *p* < 0.001),), the justification of the double rationality (*r* = 0.19; *p* < 0.001), and the practices that integrate both cultures(*r* = 0.25; *p* < 0.001).
